# Supplementary material for: Patterns of Intron Gain and Loss in Fungi
Source: PLoS Biol. 2004 Nov 30;2(12):e422. doi: 10.1371/journal.pbio.0020422 (PMC532390; doi:10.1371/journal.pbio.0020422)
Supplement: Table S1 — Also available at http://genes.mit.edu/NielsenEtAl/. (4.3 MB ZIP). [file pbio.0020422.st001.zip › NielsenEtAl/html/1327.html]

AN7588.1.NCU00519.1.MG00534.1.FG00589.1


```
 CLUSTAL W (1.82) Multiple Sequence Alignments - Introns Inserted


Sequence 1: NCU00519.1	224 aa
Sequence 2: FG00589.1	218 aa
Sequence 3: MG00534.1	251 aa
Sequence 4: AN7588.1	252 aa
Alignment Length: 253 aa
Number Identitical Residues: 134 aa
Alignment Score (without introns) 6442


MG00534.1 	MAPQAIIAPSILSADFGNLGEECSKTIGQGADWLHVDIM2DGHFVPNITFGPPVVAKIRG
NCU00519.1	------------------MGKEHQLTPNT---------R~DGHFVPNLTWGPPIVAKIRG
FG00589.1 	------------------------MTMNTP--------L2DGHFVPNITFGPPVVAAIRG
AN7588.1  	MSPPAIIAPSILSADFATLGSECSTKISEGADWLHVDIM~DGHFVPNITFGAPVVTKIRS
          	 :. :  :.:  ::. .. ... . . .  :.    .   *******:*:*.*:*: **.

MG00534.1 	HVDRPEAAHGKGTFDCHMMIAE~PKKWVKEFKAAGCDLYCFHYEAAFS--SAAEEPSATS
NCU00519.1	HVEKPTEAYGKGTFDCHMMIAE~PKKWVKEFKKAGCDLYCFHYEAAFS--TAAESPEAKS
FG00589.1 	HVDQPTEAHGRGTFDCHMMIAE0PKKWVKEFKKAGCNLYCFHYEAAFS--SAAENPEDQT
AN7588.1  	HVHRPSQPQGKGTFDCHMMIAE0PQKWVKDFKDAGCDLYCFHYEAAVSSVAAKEPADKET
          	**.:*  . *:*********** *:****:** ***:*********.*: :* * ..  :

MG00534.1 	DAKTSPRELIRYIHDQGLLAGIAIKPATPADVLYELLDSSDAAERPD~MVLVMTVEPGFG
NCU00519.1	DKKTSPKELIKYIHDQGMLAGIALKPATSVDVLWEILESEDPKERPD0MVLIMTVEPGFG
FG00589.1 	DEKTNPKALIRYIHDQGLLAGIAIKPDTSVDVLWEILENSEEKERPD0MVLVMTVYPGFG
AN7588.1  	TRKTSPKELIRFIHEEGMQAGIAIKPDTPVDVLWDILAADDEKERPD0MVLVMTVHPGFG
          	  **.*: **::**::*: ****:** *..***:::*  .:  **** ***:*** ****

MG00534.1 	GQKFMASELPKVQALRQRYPELNIEVDGGLGPGTIDQAADAGANVIVAGSAVFGAKDPAE
NCU00519.1	GQKFMASELPKVQELRKRYPELNIEVDGGLGPSTIDQAADAGANVIVAGSAVFGAQDPSE
FG00589.1 	GQKFMASELPKVQALRAKYPELNIEVDGGIGPKTIDEAADAGANVIVAGSAVFGANDPSE
AN7588.1  	GQKFMASELPKVKALREKYPDLNIEVDGGLGLGTIDQAAEAGANVIVAGSAVFGAENPGD
          	************: ** :**:********:*  ***:**:***************::*.:

MG00534.1 	VIKVLRESVQKRAGKI
NCU00519.1	VIALLREAVEKRNGKL
FG00589.1 	VIAQLRQSVDARSAK-
AN7588.1  	VIQKLRDAVNKHRKA-
          	**  **::*: :
```
